# Biosynthesis and Heterologous Expression of Cacaoidin, the First Member of the Lanthidin Family of RiPPs

**DOI:** 10.3390/antibiotics10040403

**Published:** 2021-04-08

**Authors:** Fernando Román-Hurtado, Marina Sánchez-Hidalgo, Jesús Martín, Francisco Javier Ortiz-López, Olga Genilloud

**Affiliations:** Fundación MEDINA, Avenida del Conocimiento, 34, 18016 Granada, Spain; fernando.roman@medinaandalucia.es (F.R.-H.); jesus.martin@medinaandalucia.es (J.M.); javier.ortiz@medinaandalucia.es (F.J.O.-L.); olga.genilloud@medinaandalucia.es (O.G.)

**Keywords:** cacaoidin, lanthidin, class V lanthipeptide, RiPP, *Streptomyces cacaoi*, heterologous expression

## Abstract

Cacaoidin is produced by the strain *Streptomyces cacaoi* CA-170360 and represents the first member of the new lanthidin (class V lanthipeptides) RiPP family. In this work, we describe the complete identification, cloning and heterologous expression of the cacaoidin biosynthetic gene cluster, which shows unique RiPP genes whose functions were not predicted by any bioinformatic tool. We also describe that the cacaoidin pathway is restricted to strains of the subspecies *Streptomyces cacaoi* subsp. *cacaoi* found in public genome databases, where we have also identified the presence of other putative class V lanthipeptide pathways. This is the first report on the heterologous production of a class V lanthipeptide.

## 1. Introduction

Actinomycetes are an extremely diverse group of Gram-positive, filamentous bacteria with high GC content genomes [1] considered as one of the most prolific sources for the discovery of new natural products (NPs) [2,3]. Among all the actinomycetes, the genus *Streptomyces* produces over 70–80% of the secondary metabolites with described therapeutic properties [4].

The increasing number of sequenced genomes has revealed that actinomycetes carry the genetic potential to produce many more secondary metabolites than those detected under laboratory conditions [5]. The development of bioinformatic tools to identify the presence of new secondary metabolite Biosynthetic Gene Clusters (BGCs) from genome sequences, such as antiSMASH [6] or MIBiG [7], among others [8], has permitted the development of targeted genome mining strategies directed at variants of specific families of compounds [9]. Among them, Ribosomally synthesized and Post-translationally modified Peptides (RiPPs) show more structural diversity than initially predicted from genome sequences [10]. Most RiPPs are synthesized as a longer precursor peptide, containing a C-terminal core peptide that undergoes a broad diversity of Post-Translational Modifications (PTMs) directed by the N-terminal leader peptide [11].

**Figure 1:**
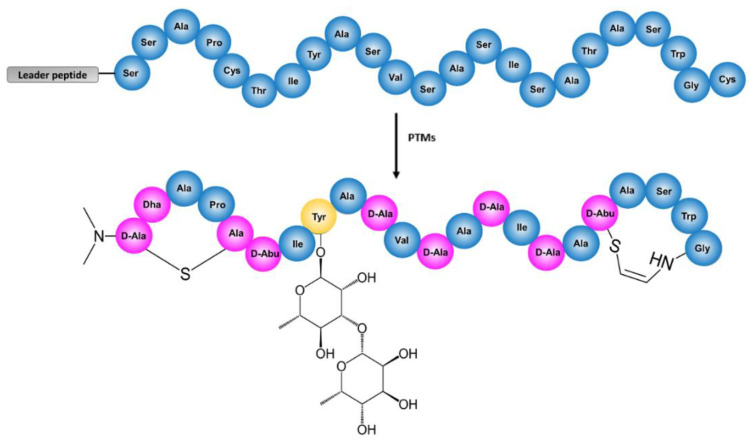
Structure of cacaoidin before (**top**) and after (**bottom**) the Post-Translational Modifications (PTMs). In the modified cacaoidin, absolute configuration for NMe2Ala(S)-1 is tentatively proposed as (2S) (equivalently, d-Ala). The amino acids modified by biosynthetic enzymes are colored in pink, the glycosylated tyrosine is colored in yellow, and the non-modified amino acids are colored in blue.

We have recently described the discovery of cacaoidin, a novel glycosylated lantibiotic produced by the strain *Streptomyces cacaoi* CA-170360 [12]. Cacaoidin represents the first reported member of lanthidins, also known as the new class V lanthipeptides, exhibiting remarkable structural features, such as a lanthionine ring and N-terminal dimethylation, restricted thus far to lanthipeptide [13] and linaridin [14,15,16] RiPP families, respectively, in an unprecedented *N*,*N*-dimethyl lanthionine system (NMe_2_Lan) [12] (Figure 1). In addition, cacaoidin contains several PTMs, some of them shared with other lanthipeptides, such as a C-terminal S-[(Z)-2-aminovinyl-3-methyl]-d-cysteine (AviMeCys) amino acid. AviMeCys is formed in lanthipeptides through an oxidative decarboxylation of the C-terminal cysteine by LanD and the resulting reactive thio–enol intermediate cyclises with a Dhb residue, yielding AviMeCys [17]. This differs from the AviCys formation in linaridins [18], which occurs between two cysteine residues and is catalyzed by LinD [19]. The co-existing structural features restricted to lanthipeptides and linaridins have supported the proposal of cacaoidin as the first reported member of the lanthidins [12]. Cacaoidin also presents other unusual structural features, such as a high number of d-amino acids and an O-glycosylated tyrosine residue carrying a non-previously reported disaccharide formed by α-l-rhamnose and β-l-6-deoxygulose [12].

Shortly after the discovery of cacaoidin, Xu et al. [20] described the novel RiPP lexapeptide as the first member of the new class V lanthipeptide. Lexapeptide is structurally similar to cacaoidin since it contains an N-terminal *N*,*N*-dimethylation, a C-terminal AviMeCys moiety and a d-Ala. According to this, and to the new RiPP classification established in a recent review [21], cacaoidin is the founding member of the class V lanthipeptides, firstly reported by our group as lanthidins.

In this work, we present the identification and analysis of the cacaoidin BGC from the genome analysis of *Streptomyces cacaoi* CA-170360, showing its distinct gene cluster organization. We show that the BGC proposed contains all the genes required for cacaoidin biosynthesis that was successfully produced via heterologous expression. To our knowledge, this is the first class V lanthipeptide to be heterologously produced. We also describe that the cacaoidin BGC is a species-specific trait within the genus *Streptomyces* and identify the presence of other class V lanthipeptide pathways in public genome databases.

## 2. Results

### 2.1. Sequencing of Streptomyces Cacaoi CA-170360 Genome and Identification of Cacaoidin BGC

*S. cacaoi* CA-170360 genome sequence was obtained with a combination of *de novo* PacBio and Illumina approaches, yielding two contigs of 5,971,081 bp and 2,704,105 bp.

The genome sequence was analyzed with antiSMASH [6], BAGEL4 [22] and PRISM [23], but none of these tools could predict the BGC responsible for cacaoidin biosynthesis, suggesting that the discovery of novel bioactive NPs using genome mining is still a challenge [10].

The C-terminal sequence of cacaoidin (Thr-Ala-Ser-Trp-Gly-Cys) was used as the query in a tBLASTn using the whole genome sequence. A 162 bp Open Reading Frame (ORF) was found to encode this sequence and helped to elucidate the final structure of the peptide [12]. Cacaoidin structural gene *caoA* encodes a 23-amino acid C-terminal core peptide (SSAPCTIYASVSASISATASWGC) following a predicted 30-amino acid N-terminal leader peptide (MGEVVEMVAGFDTYADVEELNQIAVGEAPE). Neither the leader nor the core peptide sequences showed high sequence similarity with any other lanthipeptide or linaridin (Figure S1).

**Figure 2:**
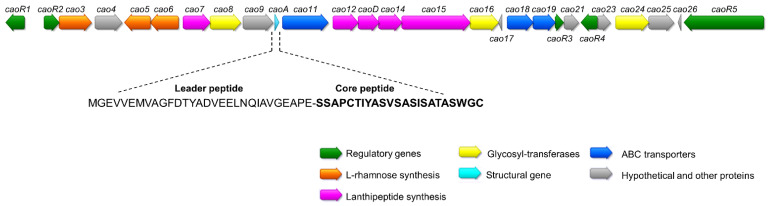
Schematic representation of the BGC of cacaoidin, where *caoA* codes for the precursor peptide. The sequences of the leader and core peptides of cacaoidin are shown.

Considering the structure of cacaoidin [12] (Figure 1), we identified a putative 30 Kb BGC (*cao* cluster) containing 27 ORFs whose proposed functions were assigned after BLAST analysis and HHpred secondary structure prediction (Figure 2, Table 1 and Table S2). Interestingly, no homologous genes of known dehydratases or cyclases commonly found in the four classes described for lanthipeptides nor in the class of linaridins could be identified in this region. Moreover, the similarity with the recently described lexapeptide BGC (*lxm*) [20] contributed as well to assigning some of the functions of the *cao* ORFs.

The *cao* BGC encodes a putative cypemycin decarboxylase CypD homologue (CaoD) containing a conserved phosphopantothenoylcysteine (PPC) synthetase/decarboxylase domain [17,24]. CaoD shares a 54.8% similarity with LxmD from the lexapeptide BGC, which is involved in the decarboxylation of C-terminal Cys residue during the formation of the AviMeCys moiety [20], a fact that supports the same role for CaoD.

The formation of lanthionine rings is accomplished by different dehydratases and cyclases depending on the lanthipeptide class [15], but no homologues were found in the *cao* cluster. In the lexapeptide biosynthesis, a three-component lanthionine synthetase formed by the standalone monofunctional proteins LxmK, LxmX and LxmY is responsible for the Ser/Thr dehydration and cyclization to form Dha and Dhb, respectively, and the installation of the lanthionine ring [20]. These proteins present a certain degree of homology with some proteins encoded in the *cao* cluster: LxmK shows a 49.6% similarity with Cao9, LxmX has a 40.5% similarity with Cao14 and LxmY shows a 44.9% similarity with Cao7. Interestingly, LxmY and Cao7 contain a HopA1 conserved domain (PFAM17914) that has been described in the HopA1 effector protein from *Pseudomonas syringae* [25].

According to these data, Cao7-9-14 would also work as a three-component lanthionine synthetase: the putative kinase Cao9 would phosphorylate Ser/Thr residues on the CaoA precursor peptide, Cao14 would remove the phosphate groups and generate dehydro-amino acids, and Cao7 would catalyze a Michael-type addition of the Cys5 thiol group to the β-carbon of Dha1, generating the lanthionine bridge, and likely the addition of previously decarboxylated-Cys23 by CaoD to Dhb18, forming AviMeCys.

The N-terminal Ala dimethylation of cypemycin, the prototypical member of linaridins [18], is carried out by the S-adenosylmethionine (SAM)-dependent methyltransferase CypM [14,19] and shows homology with the methyltransferase LxmM that catalyzes the lexapeptide N-terminal dimethylation [20]. The *cao* cluster encodes Cao4, a putative O-methyltransferase containing the conserved Methyltransf_2 domain. However, despite the low homology found between Cao4 and LxmM (26.9% similarity), both proteins share the AdoMet_MTases superfamily domain, characteristic of the SAM-dependent methyltransferases. No other CypM or LxmM homologues have been found in the genome of strain CA-170360. Cao4 shows very low homology with two types of LanS O-methyltransferases described in class I lanthipeptide clusters from actinobacteria: LanS_A_ [26] and LanS_B_ [27]. Thus, its role in the *N*,*N*-methylation remains unclear and is currently under study.

d-Amino acids provide a wide variety of properties to lanthipeptides [28] and are introduced by modifying the genetically encoded l-Ser and l-Thr into Dha and Dhb, which are subjected to a diastereoselective hydrogenation to finally incorporate d-Ala and d-Abu, respectively [15,27]. This reaction is carried out by dehydrogenases generically called LanJ [15], which are divided into zinc-dependent dehydrogenases (LanJ_A_), which can only hydrogenate Dha, and flavin-dependent dehydrogenases (LanJ_B_) able to reduce both Dha and Dhb [29,30]. Xu et al. [20] reported LxmJ as the first example of the LanJ_C_ class in lanthipeptides. LxmJ is a novel F_420_H_2_-dependent reductase that catalyzes the stereospecific reduction of Dha28 to d-Ala28 in the mature lexapeptide. Within the *cao* BGC, Cao12 shows a 58,6% similarity with LxmJ, and both proteins contain a F_420_H_2_-dependent oxidoreductase (MSMEG_4879 family) conserved domain. This suggests that Cao12 could be also involved in the incorporation of d-amino acids (four d-Ala and one d-Abu) in the cacaoidin structure. It has been demonstrated that LxmJ is unable to reduce Dhb when a S28T point mutation is introduced in LxmA. However, as it was shown for BsjJ_B_ in bicereucin biosynthesis [30], the reduction may be dependent of the position and/or sequence, so the incorporation of d-Abu in cacaoidin could also be performed by Cao12.

The disaccharide moiety *β*-6-deoxygulopyranosyl-(1→3)-α-rhamnopyranoside of cacaoidin has not been previously reported [12]. Three of the four proteins required for the synthesis of *α*-l-rhamnose (*rmlA, rmlB* and *rmlD*) (Figure S2) are encoded in the *cao* cluster. Nevertheless, a BLAST search of RmlC against CA-170360 whole genome sequence also shows the presence of a *rmlC* gene and additional *rmlA, rmlB* and *rmlD* homologous genes outside the cacaoidin cluster. Indeed, it has been reported that the *rml* genes do not have to be necessarily clustered [31]. Bleomycin, tallysomycin and zorbamycin incorporate NDP-l-gulose or NDP-6-deoxy-l-gulose to their structures, and their biosynthesis was used as reference to look for the presence of similar proteins being encoded in our genome (Figure S3) [32]. Despite no homologues being found in the cacaoidin BGC, we identified some protein homologues in the genome. However, as none of them were clustered, no conclusions could be made for the *β*-l-6-deoxy-gulose biosynthesis.

As one of the unusual structural features of cacaoidin, the disaccharide is O-linked to the aromatic ring of the tyrosine residue [12]. While serine, threonine or hydroxyproline O-glycosylation have been reported in many natural glycopeptides [33], the O-glycosylation of tyrosine in a natural product has been only reported in the mannopeptimycins, in a reaction catalyzed by the mannosyltransferases MppH and MppI [34]. The *cao* cluster lacks MppH/I homologues, but contains three glycosyltransferases (GTs) (Cao8, Cao16 and Cao24). Cao8 and Cao16 belong to the family GT-2 and show 42% identity (54% similarity) and 43% identity (52% similarity), respectively, with an UDP-Glc:alpha-d-GlcNAc-diphosphoundecaprenol beta-1,3-glucosyltransferase WfgD, which catalyzes the addition of Glc, the second sugar moiety of the O152-antigen repeating unit, to GlcNAc-pyrophosphate-undecaprenol [35]. In contrast, Cao24 belongs to the family GT-4 that has a GT4_GtfA-like domain and a conserved RfaB domain, involved in the cell wall and membrane biosynthesis [36].

Despite the presence of three GTs in the cacaoidin BGC, only two sugar moieties in the form of disaccharide are detected in the structure. The three GTs could work in a cooperative manner to achieve effective glycosylation, as it has already been proposed [37,38,39]. Based on the “inverting” or “retaining” behavior of the glycosyltransferases, we propose that both Cao8 and Cao16, belonging to the “inverting” GT-2 family, might work cooperatively to attach the α-l-rhamnose unit, while Cao24, belonging to the “retaining” GT-4 family, would incorporate the *β*-l-6-deoxygulose unit [12].

In addition to cacaoidin, only two glycosylated RiPPs produced by bacteria have been described: the group of glycocins, whose sugar moieties are linked to Cys, Ser or Thr residues by S-glycosyltransferases [40], and the class III lanthipeptide NAI-112, which carries a 6-deoxyhexose moiety N-linked to a tryptophan residue by the AplG GT [41]. However, low homologies were found between these enzymes and the GTs present in the cacaoidin cluster. Further studies will clarify the role of each GT in cacaoidin biosynthesis.

Processing of leader peptide is another key step in the post-translational modification of RiPPs [15,42,43,44,45,46]. In the cacaoidin cluster, Cao15 encodes a putative Zn-dependent peptidase belonging to the M16 peptidase family that may be involved in the leader peptide processing. Peptidases LxmP1 and LxmP2 from lexapeptide BGC also belong to the M16 peptidase family and share 25.4% and 30.4% similarity with Cao15, respectively. LxmP1 has an inactive M16 peptidase domain and LxmP2 an active domain. It has been suggested that LxmP1 and LxmP2 may form a heterodimer to remove the leader peptide from LxmA [20]. Since Cao15 harbors both the active and the inactive M16 peptidase domains, we propose Cao15 as the cacaoidin leader peptidase.

Furthermore, three ABC transporters were found in the *cao* pathway (Cao11, Cao18 and Cao19) that might be responsible for the export and self-resistance of cacaoidin, as it has been proposed in other lanthipeptides [47,48].

Gene expression in the cacaoidin cluster seems to be under the control of five different transcriptional regulators: one LuxR (CaoR1), two HTH-type XRE (CaoR2 and CaoR3), one TetR (CaoR4) and one SARP (CaoR5). XRE and TetR have been described as transcriptional repressors [49,50] while LuxR and SARP have been designated as transcriptional activators [51,52]. Further studies will clarify their role in the production of the antibiotic.

The remaining five genes identified in the *cao* cluster (*cao17*, *cao21*, *cao23*, *cao25* and *cao26*) do not have any defined functions. Cao23 belongs to the SRPBCC (START/RHO_alpha_C/PITP/Bet_v1/CoxG/CalC) superfamily of proteins [53,54]. This superfamily contains aromatase/cyclase (ARO/CYC) domains, such as those described for tetracenomycin [55] and Smu.440 [56]. However, we cannot still propose a function for Cao23.

The HHpred analysis of each ORF was also used for the detection of RiPP precursor peptide Recognition Elements (RREs) [57]. These RRE are structurally similar PqqD-related conserved precursor peptide-binding domains present in the majority of known prokaryotic RiPP modifying enzymes and are usually responsible for the leader peptide recognition [57,58,59]. However, no RREs were found, suggesting the possibility of alternative leader peptide recognition domains that are unrelated to the already known RREs [57]. As homology detection algorithms become more accurate and more sequences become available, additional RREs will be found.

### 2.2. Cloning and Heterologous Expression of Cacaoidin BGC

The strain *S. cacaoi* CA-170360 is reluctant to genetic manipulation, limiting the obtention of knockdown mutants to confirm the involvement of the *cao* gene cluster in the biosynthesis of cacaoidin. To confirm that the genes included in the *cao* cluster were sufficient for biosynthesis of the antibiotic, we cloned and heterologously expressed the cacaoidin BGC in the genetically amenable host *Streptomyces albus* J1074 [60].

We followed the CATCH method [61] to clone a 40 Kb region containing the *cao* BGC into the pCAP01 vector [62], yielding pCAO. pCAO was introduced into NEB-10-beta *E. coli* ET12567 cells via electroporation. A triparental conjugation [63] was carried out between *E. coli* ET12567/pCAO, *E. coli* ET12567/pUB307 and *S. albus* J1074 spores [60]. The transconjugants were genetically verified via PCR amplification of the genes *cao4*, *cao8* and *cao15* using specific primers described in Table S1. Five positive transconjugants, alongside the negative control (*S. albus* J1074/pCAP01) and the wild-type strain CA-170360, were grown in R2YE, MPG, FR23, YEME and KM4 for 14 days at 28 °C to confirm the production of the targeted antibiotic. After acetone extraction of the cultures, organic solvent was evaporated, and the aqueous extracts containing 20% DMSO were analyzed via LC-HRESIMS(+)-TOF and MS/MS.

**Figure 3:**
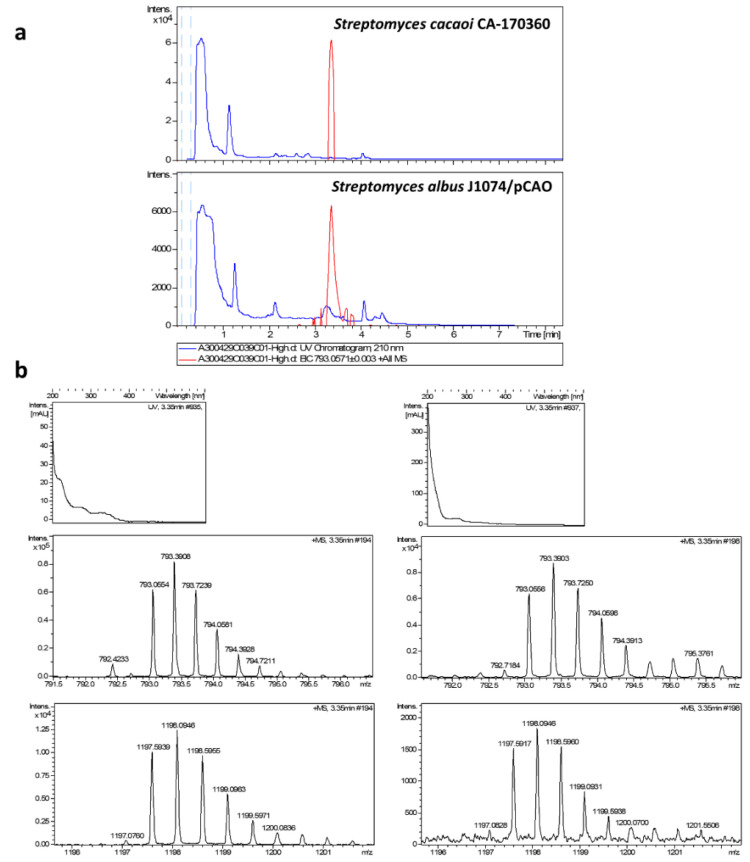
(**a**) Chromatograms of UV absorbance at 210 nm (blue trace) and extracted ion *m*/*z* = 793.0571 ± 0.003, C_107_H_162_N_24_O_32_S_2_ + NH_4_^+^ + 2H^+^ of cacaoidin (MDN-0207) from original producing strain *Streptomyces cacaoi* CA-170360 and the heterologous producing strain *Streptomyces albus* J1074 /pCAO. (**b**) Experimental UV and positive mass spectra from C_107_H_162_N_24_O_32_S_2_ + NH_4_^+^ + 2H^+^ (calculated value: 793.0571) (**top**) and C_107_H_162_N_24_O_32_S_2_ + 2NH_4_^+^ (calculated value: 1197.5952) (**bottom**) adducts from heterologous producing strain (**right**) and from original producing strain CA-170360 (**left**).

**Figure 4:**
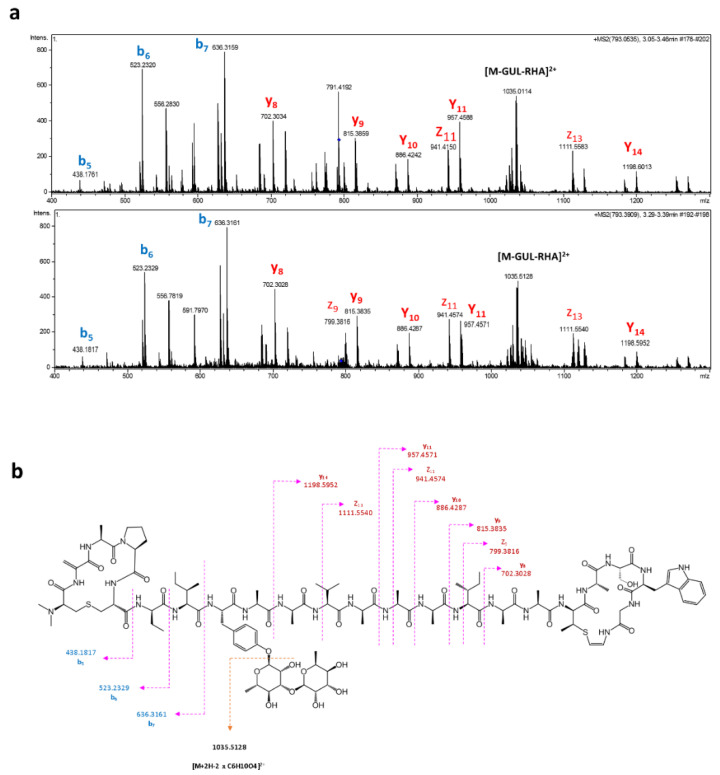
(**a**) Tandem MS/MS fragmentation pattern of C_107_H_162_N_24_O_32_S_2_ + NH_4_^+^ + 2H^+^ from strain CA-170360 (**top**) and *S. albus* J1074/pCAO (**bottom**). Annotation of b-, y- and z-ions is shown. (**b**) Key MS/MS fragmentation of [M + 2H + NH_4_]^3+^ ion.

The analysis of the extracts from pCAO transconjugants confirmed the presence of peaks at 3.35 min, coincident with the retention time of elution of cacaoidin in the wild-type strain and purified cacaoidin standards, only in R2YE medium. The perfect correlation between the UV spectrum, exact mass, isotopic distribution and MS/MS fragmentation patterns of cacaoidin standards and the components isolated from *S. albus* J1074/pCAO, undoubtedly demonstrated that they correspond to cacaoidin (Figure 3 and Figure 4). We quantified the yield of cacaoidin production in both the heterologous and the wild-type strains. *S. albus* J1074 has been largely used as heterologous host to produce a broad range of compound classes with improved yields [64]. However, the cacaoidin production yield of 0.14 mg/L in the heterologous strain grown in R2YE was reduced approximately 4-fold with respect to the 0.57 mg/L obtained in the wild-type strain CA-170360 in the same medium and fermentation format (10 mL). These results clearly confirm that the *cao* BGC cloned in pCAO contains all the genes required to ensure the biosynthesis of cacaoidin, although the production yield is dependent on the strain used.

### 2.3. Additional Lanthidin Clusters in Public Databases

**Figure 5:**
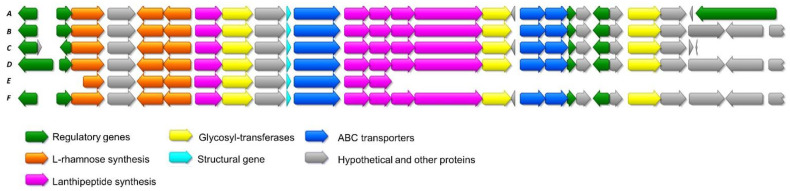
Schematic representation of the alignment of cacaoidin BGC from *Streptomyces cacaoi* CA-170360 and the highly homologous clusters found in NCBI. All of them belong as well to strains of *S. cacaoi*. A: *S. cacaoi* CA-170360; B: *S. cacaoi* subsp. *cacaoi* NBRC 12748; C: *Streptomyces* sp. NRRL S-1868; D: *Streptomyces* sp. NRRL F-5053; E: *S. cacaoi* subsp. cacaoi NRRL B-1220; F: *S. cacaoi* subsp. *cacaoi* OABC16.

To study if more lanthidin-encoding clusters can be found within actinomycetes, a BLAST search against the NCBI whole genome shotgun sequences database was performed, and sequences with a high degree of homology to cacaoidin BGC were only found in five *Streptomyces* strains, namely *Streptomyces cacaoi* subsp. *cacaoi* NRRL B-1220 (MUBL01000486), *Streptomyces* sp. NRRL F-5053 (JOHT01000009), *Streptomyces* sp. NRRL S-1868 (JOGD01000003), *Streptomyces cacaoi* subsp. *cacaoi* NBRC 12748 (BJMM01000002.1) and *Streptomyces cacaoi* subsp. *cacaoi* OABC16 (VSKT010000024) (Figure 5, Table S3).

An alignment of the precursor peptide of the cacaoidin in all homologous clusters showed that no variations in the protein sequence were found. No other cacaoidin-derived peptides or pathways were found in the databases, indicating that the cacaoidin BGC is very conserved. A phylogenetic tree generated using the neighbor-joining method and corrected with the Tamura 3-parameter algorithm [65,66] showed the close relatedness of strain *Streptomyces cacaoi* CA-170360 with the other five strains that contain the *cao* cluster, which was highly supported by the bootstrap values and contained two sequences of the type species of *S.cacaoi* subsp *cacaoi* NRRL B-1220 and NBRC 12748, as well as the strain of the same subspecies, OABC16 (Figure S4). Moreover, when the 16S rDNA sequences of the unclassified strains *Streptomyces* sp. NRRL F-5053 and *Streptomyces* sp. NRRL S-1868 were analyzed in EzBiocloud, a 100% sequence similarity was confirmed with *Streptomyces cacaoi* subsp. *cacaoi* NRRL B-1220, indicating that the cacaoidin BGC is so far limited to this specific clade of highly related strains associated to the subspecies *S. cacaoi* subsp. *cacaoi*, with no identifiable orthologs in other *Streptomyces* species. We extended the study to other members of the subspecies *S.cacaoi* subsp. *asoensis* that are clustered in a very distant phylogenetic branch from *S.cacaoi* subsp. *cacaoi* (Figure S4). We could confirm that none of the three whole genome sequences available in NCBI from strains of *S. cacaoi* subsp. *asoensis* contained any region with homology with the *cao* cluster, supporting that the *cao* BGC is a characteristic trait of the subspecies *S. cacaoi* subsp *cacaoi*. Several genome comparative studies have found other species-specific BGCs in some species of *Streptomyces*, reflecting that chemical novelty can be found at the species level and that the analysis of the genomes of closely related strains constitutes a promising approach for the identification of novel BGCs [67,68]. The occurrence of these species-specific BGCs may suggest that they were acquired recently via horizontal gene transfer as it has been shown for other pathways [69].

**Figure 6:**
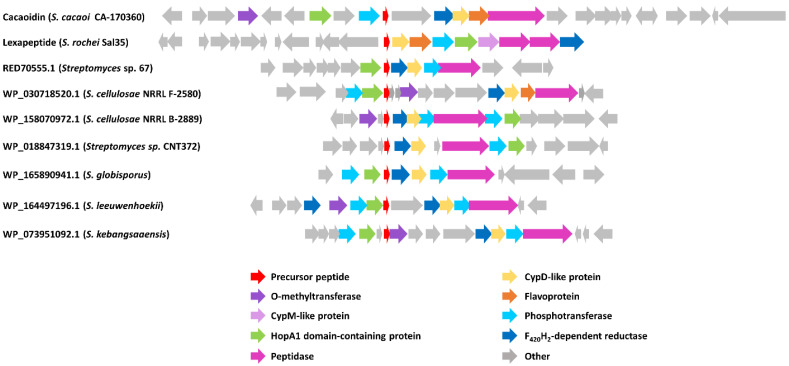
Schematic representation of cacaoidin BGC and the BGC of lexapeptide and other putative lanthidins (class V lanthipeptides). Accession numbers of each putative structural peptide are indicated.

**Figure 7:**
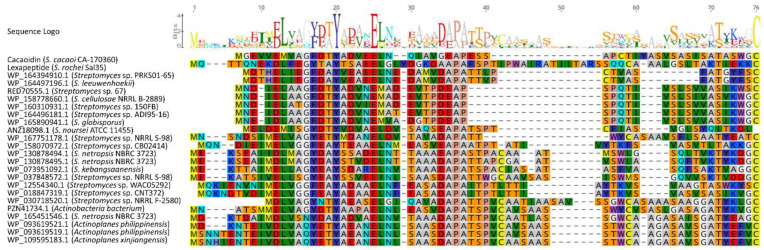
Alignment of cacaoidin and putative lanthidins (class V lanthipeptides) precursor peptides (accession numbers are shown). Sequence logo shows conserved residues.

Nevertheless, the analysis of below-threshold scores of CaoA BLAST results, together with the search of HopA1 domain-containing proteins similar to Cao7, allowed us to find some additional pathways that could encode new lanthidins (Figure 6). The alignment of the hypothetical precursor peptides shows the presence of some conserved residues that possibly could be involved in the leader peptide recognition by biosynthetic enzymes (Figure 7). In addition, the analysis of the ORFs present in all these clusters shows that all of them share a HopA1 domain-containing protein, a F_420_H_2_-dependent oxidoreductase, a CypD-related protein, a Zn-dependent or S9 peptidase and a putative phosphotransferase (Figure 6). Most of these clusters also contain an O-methyltransferase. These preliminary data suggest the existence of a minimal set of genes required to ensure the core structural features of a lanthidin and a broader distribution of potential BGCs encoding new lanthidins. These putative lanthidin BGCs are mainly present in *Streptomyces* strains, but also in two *Actinoplanes* species and another undefined member of the class *Actinobacteria*. To date, only the class V lanthipeptides cacaoidin and lexapeptide BGCs have been described. Additional members of the proposed new RiPP family will need to be isolated to confirm this hypothesis and whether class V lanthipeptides are only present in *Actinobacteria* (Figure 7).

## 3. Materials and Methods

### 3.1. Strains and Plasmids

The strain *Streptomyces cacaoi* CA-170360, from Fundación MEDINA’s Culture Collection, was isolated from the rhizosphere of *Brownanthus corallinus*, in the region of Namaqualand (South Africa). Electrocompetent NEB 10-β *E. coli* (New England BioLabs, Ipswich, MA, USA), *E. coli* ET12567 (LGC Standards, Manchester, NH, USA) and *E. coli* ET12567/pUB307 (kindly provided by José Antonio Salas) were used for plasmid transformation and intergeneric triparental conjugation. *Streptomyces albus* J1074 [60], also kindly provided by José Antonio Salas, was employed as the heterologous expression host.

Vector pCAP01, a yeast-*E. coli*-actinobacteria shuttle vector that can integrate cloned gene clusters into the genome of heterologous actinobacteria hosts due to its site-specific φC31 integrase [70], was used for the cloning of the cacaoidin BGC and was a gift from Bradley Moore (Addgene plasmid #59981; http://n2t.net/addgene:59981 (accessed on 12 January 2021); RRID: Addgene_59981).

### 3.2. DNA Extraction and Genome Mining

Genomic DNA of the strain *Streptomyces cacaoi* CA-170360 was extracted and purified as Kieser et al. described [63] from cultures on ATCC-2 liquid medium (soluble starch 20 g/L, glucose 10 g/L, NZ Amine Type E 5 g/L, meat extract 3 g/L, peptone 5 g/L, yeast extract 5 g/L, calcium carbonate 1 g/L, pH 7) grown on an orbital shaker at 28 °C, 220 rpm and 70% relative humidity.

The genome of CA-170360 was fully sequenced *de novo*, assembled and annotated by Macrogen (Seoul, Korea; http://www.macrogen.com/ (accessed on 12 January 2021)), using a combined strategy of Illumina HiSeq 2500 and PacBio RSII platforms. The PacBio long-reads were assembled with Canu (v1.7) [71]. After assembly, Illumina reads were applied for accurate genome sequence using Pilon (v1.21) [72]. To validate the accuracy of the assembly, Illumina reads were mapped to the assembly result. After mapping, the consensus sequence was generated.

### 3.3. Identification of Cacaoidin BGC

The sequence of the CA-170360 genome was analyzed with antiSMASH v4.2.0 [6], BAGEL4 [22], PRISM [23] and RiPPMiner [73] in order to find cacaoidin BGC. BLAST (Basic Local Alignment Search Tool) [74] and HHpred based on profile hidden Markov model (HMM) comparisons [75] were also employed to predict the function of each gene in the biosynthesis of the lanthipeptide. The *cao* BGC sequence is available in the National Center for Biotechnology Information (NCBI) database under accession GenBank number MT210103.

### 3.4. Cas-9 Assisted Targeting of CHromosome (CATCH) Cloning of Cacaoidin BGC

Cloning of cacaoidin BGC was performed via CATCH. The CRISPR-Cas9 endonuclease is guided by an RNA template to cleave specific DNA targets enabling larger BGC to be isolated than other techniques such as transformation-associated recombination or single-strand overlapping annealing [61].

The CATCH cloning was performed as Jian and Zhu described [76]. First, we designed the 20 nt targeted sequences flanking the BGC with the CRISPy-web tool (http://crispy.secondarymetabolites.org/ (accessed on 12 January 2021)). This computational tool design guides RNAs close to a PAM (Protospacer-Adjacent Motif) sequence ‘NGG’ [77] and we performed an overlapping PCR with the Q5 High-Fidelity polymerase from New England BioLabs (Ipswich, MA, USA), and used one specific primer within the 20 nt targeted sequence (X-sgRNA-P) and two universal primers (sgRNA-F and sgRNA-R). We obtained the sgRNA using the HiScribe T7 Quick Yield RNA synthesis kit (New England Biolabs). We also performed a PCR using the pCAP01 vector as backbone with primers containing a 20 nt sequence that anneals to the template plasmid and a 30 nt overhang overlapping with both ends of the BGC, and this PCR product was treated with DpnI (New England BioLabs) to cleave the template plasmid. Primers used for CATCH cloning are described in Table S1.

*S. cacaoi* CA-170360 was cultured in ATCC-2 for 2 days on an orbital shaker at 28 °C, 220 rpm and 70% relative humidity and the bacterial cells were embedded in low-melting agarose plugs to make the subsequent in-gel digestion with Cas9 more feasible. The plugs were treated with lysozyme, proteinase K and washing buffers, and the in-gel Cas9 digestion was performed by mixing two agarose plugs, a cleavage buffer (100 mM HEPES pH 7.5, 750 mM KCl, 0.5 mM EDTA pH 8, 50 mM MgCl2, DEPC-treated water), the sgRNAs and the Cas9 nuclease from *S. pyogenes* (New England BioLabs) and incubating at 37 °C for 2 h. After this time the agarose plugs were melted and digested with gelase and the cleavaged DNA was precipitated with ethanol and resuspended in DNase-free water.

The Cas9-digested DNA was cloned into the vector pCAP01 via Gibson Assembly using a 2× Gibson Assembly Master Mix (New England BioLabs) and incubating at 50 °C for 1 h. After the ligation, this Gibson product (pCAO) was transformed into electrocompetent NEB-10-beta *E. coli* cells. These cells were incubated in 1 mL LB broth Miller (Sigma, St. Louis, MO, USA) (37 °C, 250 rpm) without antibiotics and plated on Difco LB agar Lennox (37 °C, overnight, static) containing kanamycin (50 μg/mL).

The plasmids obtained from the selected colonies on these LB agar plates were validated by performing two independent double digestions with HindIII/NdeI and XbaI/EcoRV restriction endonucleases from New England BioLabs, which gave two different fragmentation patterns that allowed us to identify the correct pCAO construction.

### 3.5. Heterologous Expression of Cacaoidin BGC

Construction pCAO was introduced into *Streptomyces albus* J1074 host via triparental conjugation [61]. First, *E. coli* ET12567 cells were electroporated with 50 ng of the construction in a total volume of 1 µL and plated on selective LB agar plates with 50 µg/mL kanamycin. *E. coli* ET12567/pUB307 cells were also streaked on selective LB agar plates with 50 µg/mL kanamycin and 25 µg/mL chloramphenicol, which were incubated at 37 °C overnight. *E. coli* ET12567/pCAO and ET12567/pUB307 cells were collected at an OD_600_ of 0.4–0.6, washed three times with LB liquid medium without antibiotics supplementation, suspended with 100 µL LB liquid medium and mixed with 50 µL of previously activated spores (incubated at 50 °C for 10 min) of *S. albus* J1074. These mixtures were plated onto MA plates and incubated at 28 °C overnight (around 16 h). On the next day, the conjugation MA plates were overlayed with 1.5 mL of sterilized Milli-Q water containing 50 µg/mL kanamycin and 25 µg/mL nalidixic acid. These plates were incubated again at 28 °C for 3–5 days to let the exconjugants grow. After this time of incubation, five exconjugants from each *Streptomyces* heterologous host were picked and spread out on MA plates containing nalidixic acid (25 μg/mL) and kanamycin (50 μg/mL).

Five plugs from these MA plates of the recombinant strain *S. albus* J1074/pCAO were used to seed 10 mL ATCC-2 tubes, which were incubated at 28 °C for 2–3 days, 220 rpm and 70% humidity, and these cultures, together with the corresponding negative controls harboring empty pCAP01 vector, were used to inoculate 10 mL of MPG (10 g/L glucose, 20 g/L Millet meal, 20 g/L MOPS, 20 g/L pharmamedia, pH 7.0), R2YE (103 g/L sucrose, 0.25 g /L K_2_SO_4_, 10.12 g/L MgCl_2_∙6H_2_O, 10 g/L glucose, 0.1 g/L Difco Casaminoacids, 0.05 g/L KH_2_PO_4_, 2.944 g/L CaCl_2_∙2H_2_O, 3 g/L l-proline, 5.73 g/L TES buffer, 5 g/L Difco yeast extract, 5 mL 1N NaOH, 2 mL trace elements), KM4 (4 g/L glucose, 4 g/L yeast extract, 10 g/L malt extract, 2 g/L CaCO_3_) (12), YEME (3 g/L yeast extract, 3 g/L malt extract, 5 g/L peptone, 10 g/L glucose, 340 g/L sucrose) and FR23 (5 g/L glucose, 30 g/L soluble starch from potato, 20 g/L cottonseed flour, 20 g/L cane molasses, pH 7) fermentation media. These fermentations were incubated at 28 °C, 220 rpm and 70% humidity for 14 days.

### 3.6. Extraction and Detection of Cacaoidin

Cultures of the recombinant strain *S. albus* J1074/pCAO, together with the negative control harboring empty pCAP01 vector, were subjected to extraction with acetone 1:1 under continuous shaking at 220 rpm for 2 h. The organic solvent with the secondary metabolites extracted was separated from the biomass via centrifugation, the acetone was removed by a stream of nitrogen overnight and the extracts were resuspended to a final ratio of 20% DMSO/water. The resulting microbial extracts were filtered and analyzed using LC-HRESIMS(+)-TOF and MS/MS.

### 3.7. Extraction and Detection of Cacaoidin

Standard solutions of pure cacaoidin at different concentrations were prepared in 20% DMSO, and a calibration curve was obtained based on the intensity of the LC-MS peak areas (slope = 245491, Y-intercept= −6165.4, R^2^ = 0.9996). Cacaoidin concentrations from the R2YE culture extracts were determined by interpolating their LC-MS peak areas on the calibration curve (y = 245941x − 6165.4).

## 4. Conclusions

Cacaoidin is the first member of the new lanthidin (class V lanthipeptides) RiPP family characterized by the combination of structural features thus far restricted to lanthipeptide and linaridin families and encoded by a new unprecedented RiPP BGC organization that could not be predicted using antiSMASH, BAGEL4 or any other bioinformatic tool. The lack of homology with common lanthionine ring formation or double N-terminal dimethylation enzymes suggests an alternative mechanism of biosynthesis that may be similar to that of the recently described lanthidin lexapeptide. The other unusual structural features of cacaoidin, such as the high number of d-amino acids or the O-glycosylation of tyrosine are supported by the presence in the *cao* cluster of protein homologues of a F_420_H_2_-dependent oxidoreductase and three glycosyltransferases. The heterologous expression of cacaoidin BGC has demonstrated that the *cao* cluster contains all the necessary genes to biosynthesize this molecule, and future research is needed to clarify the unassigned functions of the *cao* genes.

Cacaoidin BGC was only found in the genomes of all publicly available *Streptomyces cacaoi* subsp. *cacaoi* strains and not in any other species, suggesting that this cluster may be a species-specific trait. Undoubtedly, cacaoidin BGC has an unprecedented genetic organization, completely different from any other previously described RiPP cluster. Moreover, the detection of similar putative lanthidin homologous clusters opens the door to the study of a new exciting family of RiPPs.

## Figures and Tables

**Table 1:** Closest BLAST homolog for each Open Reading Frame (ORF) in cacaoidin Biosythetic Gene Cluster (BGC).

ORF	Closest BLAST Homolog	%Identity/ Similarity	Conserved Domains	Possible Function
*caoR1*	DNA-binding response regulator [*Streptomyces cacaoi* subsp. *cacaoi*]	100/100	CitB (NarL/FixJ family, contains REC and HTH domains); HTH_LUXR	Positive regulator
*caoR2*	Helix-turn-helix domain-containing protein [*Streptomyces cacaoi*]	98.44/100	HTH_XRE superfamily	Negative regulator
*cao3*	Hypothetical protein SCA03_05120 [*Streptomyces cacaoi* subsp. *cacaoi*]	97.81/100	RmlD_sub_bind	l-rhamnose synthesis
*cao4*	Methyltransferase [*Streptomyces cacaoi*]	100/100	Methyltrans_2 (O-methyltransferase)	N-terminal dimethylation
*cao5*	GDP-mannose 4,6-dehydratase [*Streptomyces cacaoi*]	98.16/100	dTDP_GD_SDR-e (dDTP-d-glucose 4,6-dehydratase)	l-rhamnose biosynthesis
*cao6*	MULTISPECIES: glucose-1-phosphate thymidylyltransferase [*Streptomyces*]	100/100	RmlA_long (glucose-1-phosphate thymidylyltransferase)	l-rhamnose biosynthesis
*cao7*	Hypothetical protein [*Streptomyces cacaoi*]	100/100	HopA1 superfamily	Unknown
*cao8*	Glycosyltransferase family 2 protein [*Streptomyces* sp. NRRL S-1868]	99.48/100	Glycos_transf_2	Glycosylation
*cao9*	Phosphotransferase [*Streptomyces* sp. NRRL S-1868]	100/100	PKc_like (protein kinase catalytic domain)	Unknown
*caoA*	Hypothetical protein SCA03_05190 [*Streptomyces cacaoi* subsp. *cacaoi*]	100/100	No putative conserved domains detected	Structural gene
*cao11*	ABC transporter ATP-binding protein [*Streptomyces* sp. NRRL F-5053]	99.66/100	MdlB (ABC-type multidrug transport system, ATPase and permease component)	Cacaoidin biosynthesis
*cao12*	MULTISPECIES: LLM class flavin-dependent oxidoreductase [*Streptomyces*]	100/100	SsuD (Flavin-dependent oxidoreductase)	Cacaoidin biosynthesis
*caoD*	Hypothetical protein SCA03_05220 [*Streptomyces cacaoi* subsp. *cacaoi*]	99.63/100	PRK05579 (bifunctional phosphopantothenoylcysteine decarboxylase/phosphopantothenate synthase)	AviMeCys biosynthesis
*cao14*	MULTISPECIES: hypothetical protein [unclassified *Streptomyces*]	99.34/100	No putative conserved domains detected	Unknown
*cao15*	Hypothetical protein [*Streptomyces* sp. NHF165]	99.42/100	PqqL (predicted Zn-dependent peptidase)	Leader peptide cleavage
*cao16*	Glycosyltransferase family 2 protein [*Streptomyces cacaoi*]	100/100	Glycos_transf_2	Glycosylation
*cao17*	Hypothetical protein [*Streptomyces cacaoi*]	91.7/100	No putative conserved domains detected	Unknown
*cao18*	ABC transporter ATP-binding protein [*Streptomyces* sp. NRRL F-5053]	100/100	CcmA (ABC-type multidrug transport system)	Cacaoidin biosynthesis
*cao19*	MULTISPECIES: ABC transporter permease [*Streptomyces*]	99.65/100	ABC2_membrane_3 (ABC-2 family transporter protein)	Cacaoidin biosynthesis
*caoR3*	Hypothetical protein SCA03_05280 [*Streptomyces cacaoi* subsp. *cacaoi*]	100/100	HTH_XRE domain	Negative regulator
*cao21*	Hypothetical protein [*Streptomyces* sp. NRRL S-1868]	100/100	No putative conserved domains detected	Unknown
*caoR4*	TetR/AcrR family transcriptional regulator [*Streptomyces cacaoi*]	100/100	AcrR (DNA-binding transcriptional regulator)	Negative regulator
*cao23*	Hypothetical protein SCA03_05310 [*Streptomyces cacaoi* subsp. *cacaoi*]	99.39/100	SRPBCC superfamily	Unknown
*cao24*	MULTISPECIES: glycosyltransferase family 4 protein [*Streptomyces*]	100/100	GT4_AmsD-like	Glycosylation
*cao25*	Hypothetical protein [*Streptomyces cacaoi*]	99.06/100	No putative conserved domains detected	Unknown
*cao26*	No homologues found	-	-	Unknown
*caoR5*	Tetratricopeptide repeat protein [*Streptomyces* sp. NRRL S-1868]	99.31/100	BTAD (Bacterial Transcriptional Activation)	Positive regulator

## Data Availability

The data presented in this study are openly available in NCBI, GenBank reference number [MT210103]. Publicly available datasets were analyzed in this study. These data can be found in NCBI, GenBank reference numbers [MUBL00000000.1, VSKT00000000.1, JOGD00000000.1, JOHT00000000.1 and BJMM00000000.1].

## Supplementary Materials

The following are available online at https://www.mdpi.com/article/10.3390/antibiotics10040403/s1, Table S1: Primers used for the cloning and checking of the *cao* biosynthetic gene cluster, Table S2: Closest HHpred homolog for each ORF in cacaoidin BGC, Table S3: BLAST homology between the ORFs in cacaoidin BGC and NCBI found clusters, Figure S1: Alignment the structural amino acidic sequence of cacaoidin with the sequences of other already known lanthipeptides and linaridins, Figure S2: Schematic presentation of the biosynthesis of dTDP-l-rhamnose from d-glucose, Figure S3: Proposed pathway for the *β*-l-6-deoxy-gulose sugar biosynthesis for the BLM, TLM and ZMB compounds, Figure S4: Neighbor-joining tree built with Mega X based on nearly complete 16S rRNA gene sequences of CA-170360, the 50 closest type strains of the genus *Streptomyces* and three strains of *Streptomyces cacaoi* subsp. *asoensis*.
