# Supplementary material for: Biosynthesis and Heterologous Expression of Cacaoidin, the First Member of the Lanthidin Family of RiPPs
Source: Antibiotics (Basel). 2021 Apr 8;10(4):403. doi: 10.3390/antibiotics10040403 (PMC8068269; doi:10.3390/antibiotics10040403)
Supplement: Supplementary file 1 [file antibiotics-10-00403-s001.pdf]

antibiotics
MDPI

Supplementary Materials

Biosynthesis and Heterologous Expression of Cacaoidin, the First Member of The Lanthidin Family of Ripps

Fernando Román-Hurtado, Marina Sánchez-Hidalgo*, Jesús Martín, Francisco Javier Ortiz-López and Olga Genilloud

Fundación MEDINA, Avenida del Conocimiento, 34, 18016 Granada, Spain; fernando.roman@medinaandalucia.es (F.R-H), jesus.martin@medinaandalucia.es (J.M), javier.ortiz@medinaandalucia.es (F.J.O-L), olga.genilloud@medinaandalucia.es (O.G)
* Correspondence: marina.sanchez@medinaandalucia.es

Tables

Table S1. Primers used for the cloning and checking of the *cao* biosynthetic gene cluster.

Oligonucleotide	Sequence (5'-3')
Glyco1-sgRNA	TAATACGACTCACTATAGGACGACTCACGTGTCAAAGAGTTTTAGAGCTAGAAATAGCAA
Glyco2-sgRNA	TAATACGACTCACTATAGGGGCGAGATGCCATTCCAAGGTTTTAGAGCTAGAAATAGCAA
sgRNA-F	GTTTTAGAGCTAGAAATAGCAAGTTAAAATAAGGCTAGTC
sgRNA-R	AAAAGCACCGACTCGGTGCCACTTTTTCAAGTTGATAACGGACTAGCCTTATTTTAACT
pCAP01-Glyco-F	CGTGCGGTGGACCGCGCCGTGACCCCCTTGTCGAGACTTGAGGTACCTGT
pCAP01-Glyco-R	GGGCCGGGTTCCAGCCGGTGATGCCGTCTTTCGAGGTTACTAGTCGATCT
cao4 check-F	CGCGCACTGACCGTCTG
cao4 check-R	CTGCTGCCGGACTGACC
cao8 check-F	CCAACATCGACCCGGACC
cao8 check-R	GTCACGCCGCCACACTC
cao15 check-F	TGGGGCATCTGATCAAGGAG
cao15 check-R	TCGCGCAGTACCTCGGTT

Table S2. Closest HHPred homolog for each ORF in cacaoidin BGC. HHPred probabilities give the most relevant representation of significance with ≥90% usually being considered a true positive.

	Closest HHpred Homolog	Probability	E-value
caoR1	Response regulator protein VraR; *Enterococcus faecium*, LiaR, response regulator	99.96	1.5e-25
caoR2	Methylphosphonate synthase	98.06	0.00017
cao3	dTDP-4-dehydrorhamnose reductase, RfbD ortholog	99.97	8e-28
cao4	O-methyltransferase family protein	100	6.2e-37
cao5	Adenosylhomocysteinase (E.C.3.3.1.1, 1.8.1.7	100	1.9e-37
cao6	Bifunctional protein Glm	100	7.8e-37
cao7	Type III effector HopA1	100	3.4e-39
cao8	Putative glycosyltransferase protein	99.94	2.4e-25
cao9	5-methylthioribose kinase (E.C.2.7.1.100)	99.9	2.3e-21
caoA	Myocyte-specific enhancer factor 2A	25.67	67
cao11	Lipid A export ATP-binding/permease protein	100	1.3e-89
cao12	Bacterial luciferase, 1,2-ethanediol; monooxygenase, flavoprotein	100	1e-41
caoD	MrsD protein	100	7.5e-32
cao14	Spectinomycin phosphotransferase; protein kinase, aminoglycoside phosphotransferase, antibiotic;	99.76	5.4e-17
cao15	Insulinase family protein; Protease, M16 Family, Processing Protease	100	3e-65
lcao16	Glycosyltransferase	99.95	3.5e-26
cao17	Light-harvesting protein B-875 alpha chain	54.44	10
cao18	ABC transporter ATP-binding protein	100	2.5e-51
cao19	ATP-binding cassette sub-family G member	99.93	7.2e-23
caoR3	Putative transposon-related DNA-binding protein	99.56	8.8e-14
cao21	Ubiquinol-cytochrome c reductase iron-sulfur subunit	59.39	56
caoR4	TRANSCRIPTIONAL REGULATORY PROTEIN (PROBABLY TETR-FAMILY); tetR family of transcriptional regulator	99.94	3.3e-24
cao23	Conserved protein; Structural genomics, unknown function, ligand;	99.89	7.2e-20
cao24	Glycosyl transferase (E.C.2.4.1.57)	100	5.8e-38
cao25	TarM; Glycosyltransferase, GT-A, Wall teichoic acid	97.73	0.0025
cao26	ABC transporter ATP-binding protein (E.C.3.6.3.-)	87.92	1.5
caoR5	Tetratricopeptide repeat sensor PH0952	100	1.2e-36

Table S3. BLAST homology between the ORFs in cacaoidin BGC and NCBI found clusters.

	***Streptomyces cacaoi* subsp. *cacaoi* NBRC 12748**		***Streptomyces* sp. NRRL F-5053**		***Streptomyces* sp. NRRL S-1868**		***Streptomyces cacaoi* subsp. *cacaoi* NRRL B-1220**		***Streptomyces cacaoi* subsp. *cacaoi* OABC16**	
	**% Identity**	**% Similarity**	**% Identity**	**% Similarity**	**% Identity**	**% Similarity**	**% Identity**	**% Similarity**	**% Identity**	**% Similarity**
caoR1	100	100	99.2	99.6	100	100	-	-	99.6	100
caoR2	97.9	99	98.1	98.1	34.4	40.6	-	-	97.9	99
cao3	97.8	98.1	99	99	96.6	97.1	99.6	100	98.1	98.3
cao4	99.1	99.7	99.7	100	99.1	99.7	99.1	99.7	100	100
cao5	97.9	98.8	97.5	98.2	98.2	98.8	97.9	98.8	98.2	98.8
cao6	100	100	99.7	100	100	100	100	100	99.2	99.7
cao7	99.7	99.7	98.9	99.4	99.1	99.4	99.4	99.7	99.7	100
cao8	98.7	99.5	99.5	99.5	99.5	99.7	98.7	99.5	99.2	99.5
cao9	99.7	99.7	99.7	100	100	100	99.7	99.7	99.5	100
caoA	100	100	100	100	100	100	100	100	100	100
cao11	99.1	100	99.7	100	99.1	100	99.1	100	99.5	100
cao12	99.7	100	100	100	99.7	100	99.7	100	99.7	100
caoD	99.6	100	92.1	92.1	93.3	93.3	99.6	100	94.6	95.7
cao14	99.3	99.3	99.3	99.3	98.7	99	-	-	99	99
cao15	98.6	99	98.6	98.8	99	99.3	-	-	98.4	98.8
cao16	100	100	99.7	99.7	99.4	99.4	-	-	100	100
cao17	-	-	-	-	100	100	-	-	100	100
cao18	99.1	99.1	100	100	99.7	99.7	-	-	99.4	99.4
cao19	99.7	99.7	99.7	99.7	99.7	99.7	-	-	99.3	99.7
caoR3	100	100	100	100	99	100	-	-	99.1	100
cao21	99.5	99.5	99.5	99.5	100	100	-	-	98.9	99.5
caoR4	100	100	100	100	100	100	-	-	100	100
cao23	99.4	99.4	100	100	98.8	98.8	-	-	99.4	100
cao24	100	100	100	100	100	100	-	-	100	100
cao25	99.1	99.1	96.9	97.8	98.8	99.1	-	-	98.4	98.8
cao26	-	-	-	-	-	-	-	-	-	-
caoR5	-	-	-	-	-	-		-		-

Figures

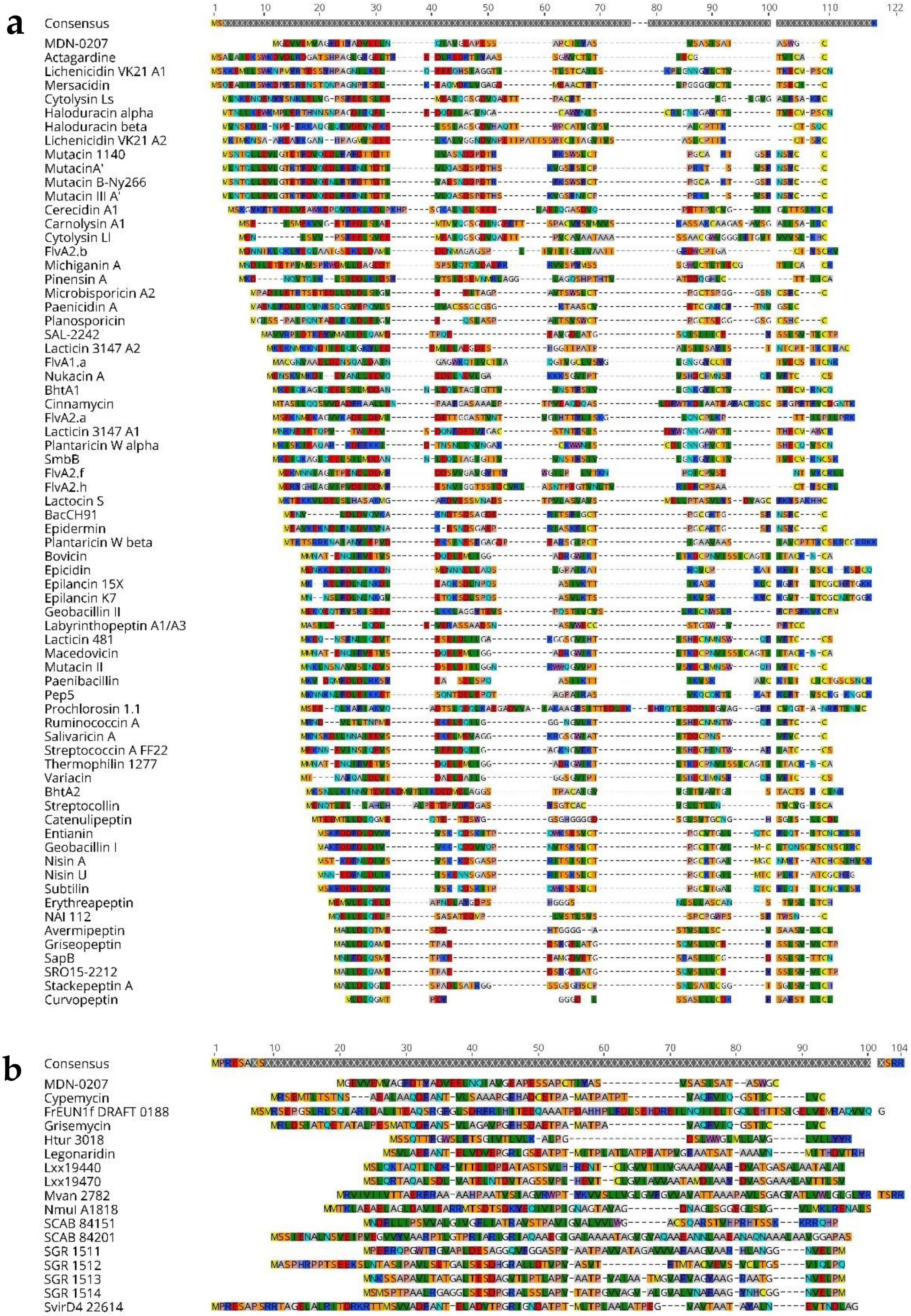

Figure S1. Alignment of the structural amino acid sequence (leader peptide + core) of cacaoidin (MDN-0207) with the sequences of other already known lanthipeptides **(a)** and linaridins **(b).**

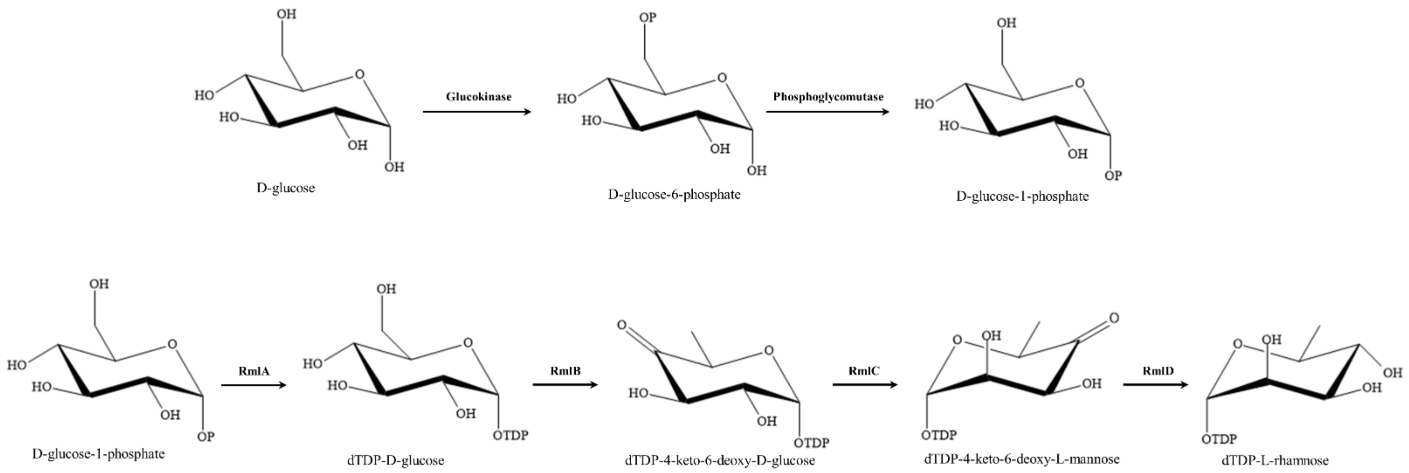

Figure S2. Schematic representation of the biosynthesis of dTDP-L-rhamnose from D-glucose.

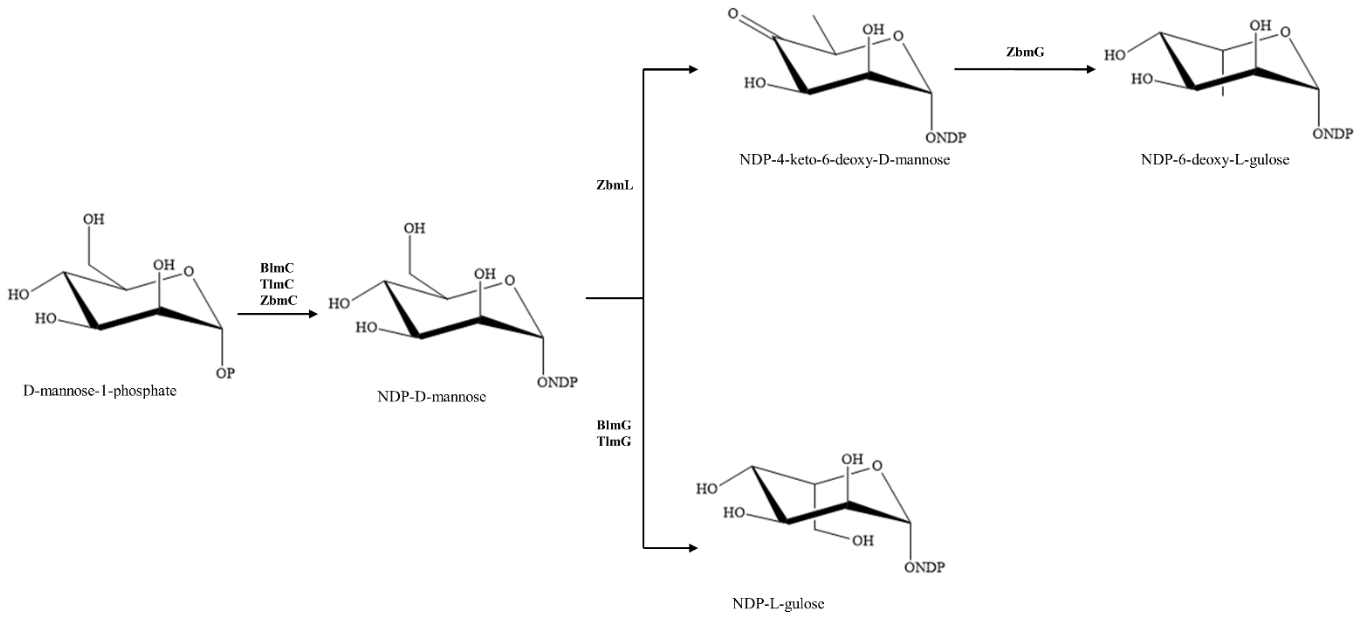

Figure S3. Proposed pathway for the *β*-L-6-deoxy-gulose sugar biosynthesis for the BLM, TLM and ZMB compounds.

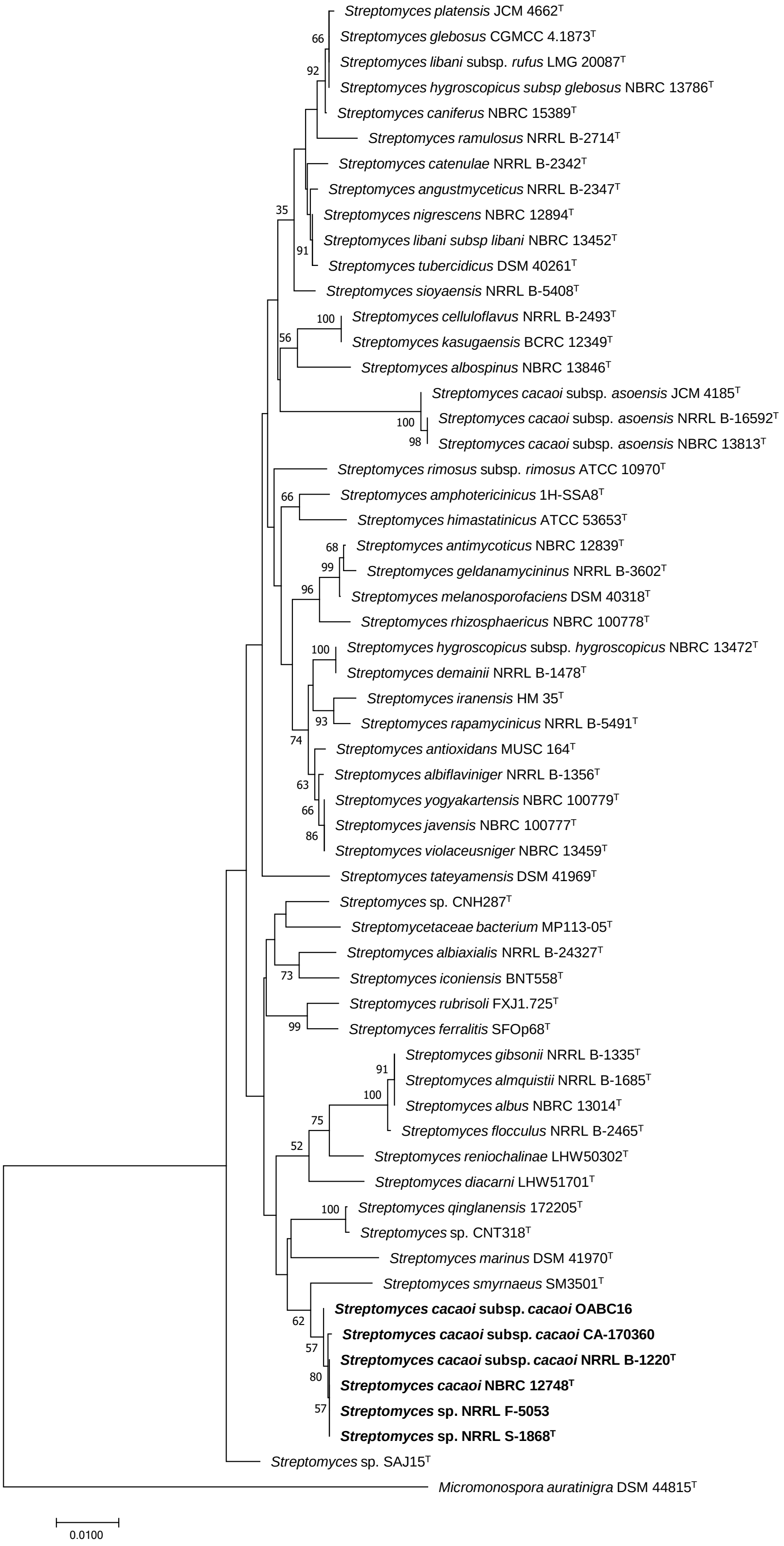

Figure S4. Neighbor-joining tree built with Mega X based on nearly complete 16S rRNA gene sequences of CA-170360, the 50 closest type strains of the genus *Streptomyces* and three strains of *Streptomyces cacaoi* subsp. *asoensis*. *Micromonospora auratinigra* DSM 44815 (T) was used as an outgroup. The numbers at the nodes indicate the bootstrap value (%) based on NJ analysis of 1000 replicates; only values higher than 50% are shown.
